# Laparoscopic nephrectomy for benign non functioning kidneys

**DOI:** 10.4103/0972-9941.19261

**Published:** 2005-10

**Authors:** Narmada P. Gupta, Gagan Gautam

**Affiliations:** Department of Urology, All India Institute of Medical Sciences, New Delhi, India

**Keywords:** nephrectomy, kidney, benign, retroperitoneoscopy

## Abstract

Laparoscopic nephrectomy has been established as the standard of care for the management of benign non-functioning kidneys and has gained worldwide popularity over the past decade. In this article, we have reviewed the current literature to elucidate the indications, contraindications, surgical techniques, results and complications of laparoscopic nephrectomy.

## INTRODUCTION

Laparoscopic urology has rapidly evolved since the mid-1990s through advances in video technology and instrumentation design and is currently a useful alternative to treat complex surgical diseases encompassing ablative as well as reconstructive urology.

In 1990, Clayman et al performed the first laparoscopic nephrectomy for a 3 cm renal mass in an elderly patient. This accomplishment represents one of the milestones in minimally invasive surgery because it provided the solution for removing a large solid organ without the need for an incision.[1]

Since this report, many institutions have verified the utility of laparoscopic approach to address the diseases of the kidney. Laparoscopic nephrectomy has proven to be beneficial as compared to open surgery in terms of lesser post operative pain, a shorter hospital stay, reduced convalescence, and a more rapid return to full activity.[2,3]

### Indications

Laparoscopic simple nephrectomy is indicated in the treatment of most benign renal diseases in which permanent loss of renal function has occurred. Indications include chronic pyelonephritis, obstructive or reflux nephropathy, renal tuberculosis, multicystic dysplastic kidney, renovascular hypertension, acquired renal cystic disease in dialysis patients, nephrosclerosis, symptomatic patients with autosomal dominant polycystic kidney disease and post kidney transplantation hypertension.[4]

### Contraindications

A prior abdominal surgery with the formation of intra-abdominal adhesions can pose a technical difficulty during insufflation, trocar placement and dissection and such a patient requires thorough preoperative planning with regards to the approach and feasibility of laparoscopic surgery. In patients with intraperitoneal adhesions, a retroperitoneoscopic approach may provide a virginal plane of dissection resulting in a decreased chance of complications. An initial access site away from the site of a previous incision or an open trocar placement may be required to achieve a similar end.[5]

Morbid obesity may create a challenge to the surgeon due to difficulty in retracting bulky fatty tissue and identifying proper planes during dissection. The increased distance to the operative field may require a change in the traditional sites of trocar insertion. Additional trocars may also be required for the introduction of retractors and additional instruments in the field.[6] Although obesity is not considered a contraindication for laparoscopic surgery and, indeed, these patients may benefit the most from avoiding a debilitating open surgery, the chances of complications and conversion to open surgery are significantly more in this group of patients.[7]

Intestinal obstruction and paralytic ileus can markedly increase the risk of injury to the bowel by reducing the working space to a large extent and should be regarded as a contraindication to the laparoscopic approach. Uncorrected coagulopathy, untreated infection and hypovolemic shock constitute the other contraindications for laparoscopoic nephrectomy.[8] Xanthogranulomatous pyelonephritis (XGPN) and renal tuberculosis may be associated dense perinephric adhesions with a concomitantly increased complication and conversion rate and may be considered as contraindications to laparoscopic approach except in the hands of experts.[9]

Severe cardiac or pulmonary disease may place the patient at risk for complications due to the pneumoperitoneum, which can compromise ventilation and limit venous return. Patients with chronic obstructive pulmonary disease may not be able to compensate for hypercarbia induced by the pneumoperitoneum and may require lower insufflation pressures or conversion to open surgery.[10]

### Patient evaluation and preparation

A patient planned for laparoscopic nephrectomy must be informed regarding the possible complications including adjacent organ injury and unrecognized bowel injury and consent should also be taken for a possible need for conversion to open surgery in case of inability to successfully complete the surgical procedure laparoscopically.[11]

Preoperative evaluation must include a careful history and detailed physical examination in order to determine any contraindications to laparoscopic surgery or conditions that may alter the laparoscopic approach. Prior abdominal surgery may alter the choice between transperitoneal or retroperitoneal approaches, patient positioning, and placement site of trocars.[12] Laboratory and imaging studies are obtained as indicated. An electrocardiogram and a chest radiograph are obtained before surgery. Pulmonary function studies are performed only in those patients with known respiratory disease or those at high risk based on the history and physical examination. All patients should have their blood typed and cross-matched blood should be available if the need arises. Mechanical bowel preparation with polyethylene glycol (Peglac®) is usually done on the preoperative day and the patient is put on clear fluids in case a transperitoneal approach for laparoscopic nephrectomy is planned. A retroperitoneoscopic approach does not require a formal bowel preparation apart from an enema administered a night before surgery.

Oral anticoagulants and anti-platelet drugs need to be stopped at least a week prior to surgery and if required the patient can be put on short acting agents like heparin whose effects can easily be reversed by using protamine. These patients also require a screening coagulation profile which may need to be corrected before taking up the patient for surgery.

### Surgical techniques

There are two basic laparoscopic approaches for simple nephrectomy: retroperitoneal and transperitoneal. A third approach, the hand assisted technique attempts to bridge the gap between laparoscopic and open surgery and may help surgeons without advanced laparoscopic training. Hand-assisted laparoscopic surgery permits tactile feedback and allows the hand to assist with dissection, retraction, extraction and rapid control of bleeding if required. The site of insertion of the device can subsequently also be used for the extraction of the specimen. This technique may be of benefit in patients with dense perinephric adhesions as in renal tuberculosis and XGPN or in those with a prior history of surgery. However, this technique requires a significantly longer incision and significantly adds to the cost of the procedure in view of the disposable nature of the hand assist devices.[13]

For laparoscopic nephrectomy, the patient is initially positioned supine for IV access, induction of general anesthesia and endotracheal intubation. A bladder catheter and nasogastric tube is placed for decompression of the bladder and stomach prior to insufflation. The subsequent steps and positioning of the patient depends on the approach for the procedure.

### Retroperitoneoscopic nephrectomy

**Figure 1:**
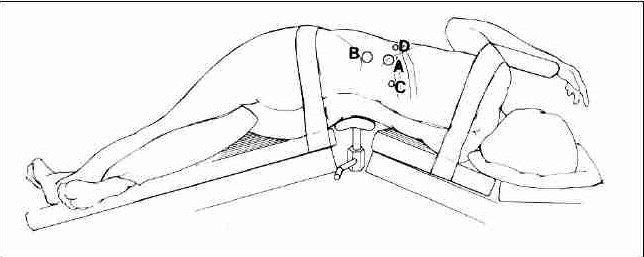
Patient's position with sites of trocar site insertion for retroperitoneoscopic nephrectomy. A: primary port, B-D: secondary ports

The patient is placed in the lateral flank position with elevation of the kidney bridge. Further, the table may be tilted anteriorly to allow the peritoneum and bowel to fall away from the proposed port site. The primary port (Figure 1-A) is placed using a 1.5-cm incision, 2 cm below and posterior to the tip of the 12^th^ rib in the posterior axillary line, deepened down to the thoracolumbar fascia (Figure 1). A retroperitoneal space is created using an indigenous or a commercially available PDB balloon®. Two or three secondary ports are inserted under laparoscopic vision or finger guided to avoid transgression of the peritoneum (Figure 1 B-D). The hilar vessels are dissected first and divided. The ureter is dissected and divided. The kidney is mobilized all round and delivered intact by extending a port or by joining two ports. Alternatively, the specimen may be removed piecemeal after morcellation within a plastic bag. A 14-F tube drain is left behind in the retroperitoneal space through the 5-mm port site at the discretion of the surgeon.[14]

The retroperitoneal approach mimics traditional open surgery in that kidney is approached without entry into the peritoneal cavity thereby providing an advantage in patients with a history of multiple abdominal surgical procedures or peritonitis. It is therefore imperative that the laparoscopic surgeon should be familiar with both transperitoneal and retroperitoneal approaches.[15] Retroperitoneal approach also provides direct access to the hilum resulting in early vascular control and making the subsequent dissection easier.

The disadvantages of the retroperitoneal approach include a steeper learning curve in view of an absence of traditional landmarks and a limited working space that can result in difficulty with orientation, visualization, trocar spacing and organ entrapment. Even though this provides an extraperitoneal operation, injury can occur to intra-abdominal organs, and a hernia can be created during balloon dilatation of the extraperitoneal space. The retroperitoneal approach results in complication rates, pain medication requirements, length of hospital stay and times to return to normal activity after surgery similar to those of transperitoneal route.[16]

### Transperitoneal laparoscopic nephrectomy

Traditionally, the transperitoneal route is the method used to perform laparoscopic surgery. It affords an optimal working space and facilitates orientation by providing readily identifiable anatomic landmarks.

**Figure 2:**
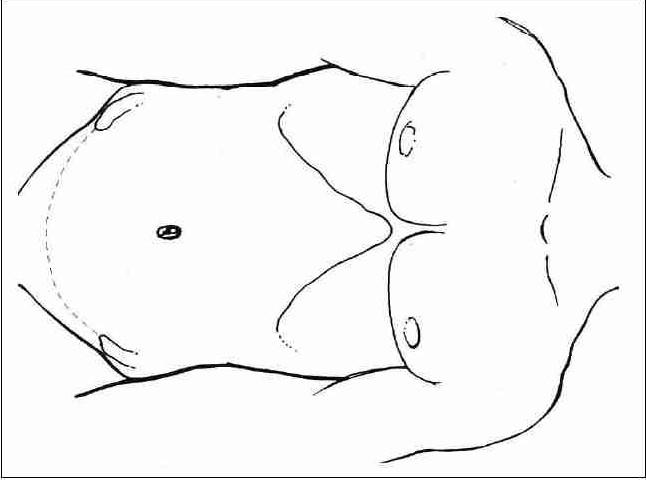
Sites of trocar placement for transperitoneal laparoscopic nephrectomy (A: 10 mm camera port, B: 10 mm, C: 5 mm)

The initial trocar may be placed after insufflating the abdomen using a Veress needle or by the open (Hasson's) technique. With the patient in the flank position, the preferred site for initial port placement is at the level of the umbilicus, lateral to the ipsilateral rectus muscle. Remaining trocars are placed under direct vision. A three-trocar technique is usually utilized to complete the dissection (Figure 2). Additional trocars for retraction may be needed to complete the hilar dissection or assist with organ entrapment.

For a left nephrectomy, the white line of Toldt is incised from the level of the iliac vessels to above the spleen including the lienocolic ligament. During a right-sided nephrectomy, the peritoneal incision is carried cephalad, above the hepatic flexure including the right triangular and right anterior coronary ligaments. Medial traction on the colon reveals colorenal attachments that must be divided to complete the colon dissection. Adequate mobilization of the colon reveals the psoas muscle followed by the gonadal vessels and the ureter. The ureter is elevated and followed proximally to the lower pole and hilum of the kidney. The ureter is not divided at this time because it can be used to help elevate the kidney and identify the hilar vessels which are clipped and divided individually after a meticulous hilar dissection.

Once the hilar vessels have been divided, the dissection continues posteriorly and superiorly to the upper pole and the adrenal gland is preserved. The ureter is divided and the kidney is removed intact by extending a 10 mm port or morcellated in an entrapment sac. The muscle layer of the 10 mm trocar sites is closed with 2-0 vicryl sutures.

### Postoperative management

The nasogastric tube is removed at the conclusion of the procedure. The patient can begin oral diet as tolerated after the bowel sounds return. The Foley catheter is removed once the patient is ambulating and a drain (if inserted) can be removed when the output is less than 50 ml in 24 hrs. The patient is discharged when tolerating a diet. Unrestricted activity can usually be resumed according to the patient's comfort.

## RESULTS

Several large series have demonstrated that laparoscopic nephrectomy compares favorably with open surgery with regards to decreased pain and shorter convalescence. Hospital stay has been decreased by 50%, and the time to full convalescence has been reported to be markedly less than with open removal. With growing expertise and experience current operative times have decreased dramatically and are comparable to those in the open group.[14,16].There is controversy in literature concerning the selection between transperitoneal and retroperitoneal laparoscopic access for nephrectomy. The chosen technique usually depends on the surgeon's own choice as a result of his expertise and training.

The retroperitoneal access allows a procedure without manipulation of the intraperitoneal organs, reducing the risk of direct and indirect damage to these structures. In addition to reducing the incidence of adynamic ileus and adhesions, the retroperitoneal access keeps the peritoneal cavity isolated from urinary fistulas and post operative infectious processes.[17] This access also enables early control of the renal pedicle, which can result in a major advantage in case of inflammatory renal disease. Hemal et al reported that the dissection and initial ligation of the renal pedicle in retroperitoneoscopic nephrectomy decreases the index of complication and the conversion rate.[18]

In a recent series, we compared a series of 351 retroperitoneoscopic nephrectomies with 83 open procedures. Mean operative time was longer in the retroperitoneoscopic group as compared to the open group (98 min v/s 70 min). However, mean blood loss (65 v/s 110 ml), complication rate (13.3% v/s 25.3%), hospital stay (3 days v/s 5 days) and convalescence (11 days v/s 28 days) were significantly less in the retroperitoneoscopic group. Moreover, with growing experience, the operating time and complications were found to decrease. It was concluded that retroperitoneoscopic nephrectomy should be regarded as the standard of care in the treatment of benign non functioning kidneys unless specific contraindications exist.[19]

### Complications

Laparoscopic nephrectomy shares several potential risks with open surgery; however there are differences in the type and presentation of these complications. Complications can arise at each step of the procedure. Access related problems like solid organ injury, bowel injury, abdominal wall hematoma and epigastric vessel injuries have been reported. Bleeding related complications can be reduced by careful inspection of the operative field prior to exit after lowering the intra-abdominal pressure and by careful surgical technique.

Volume overload is also a potential problem which is more pronounced in patients with diminished cardiac reserve and those undergoing prolonged procedures. Additionally reported common complications include incisional hernia, transient thigh numbness, prolonged ileus, pulmonary embolus, pneumonia, brachial nerve injury and unrecognized bowel injury.[20]

The combined incidence of bowel injury is 1.3 in 1000 cases and as many as 69% of those are not recognized intraoperatively.[21] Patients with unrecognized bowel injury after laparoscopy typically present with persistent and increased trocar site pain at the site closest to the bowel injury. Later signs and symptoms include nausea, diarrhea, anorexia, low grade fever, persistent bowel sounds and a low or normal WBC count. The patient's condition can rapidly deteriorate to hemodynamic instability and death if the injury is not quickly recognized and treated. CT is the initial diagnostic modality of choice and open exploration is usually required to evacuate bowel spillage and perform the necessary repair.[22]

The overall complication rate has ranged from 6–17% in contemporary series with minor complications forming the predominant portion. Conversion rates have also ranged from 5–12% with a large contemporary series by Gupta et al reporting the need for conversion in 22 out of 351 retroperitoneoscopic nephrectomies yielding a conversion rate of 6.3% with most of those occurring in the first 100 cases.[3,16,19]

In a multi-center analysis of 153 patients undergoing laparoscopic nephrectomy for benign conditions, Gill et al reported complications in 19 (12%) patients with most of the complications (n=12) occurring in the first 20 cases performed. Five patients required conversion to open surgery, out of which 4 cases were amongst the first 20 that were performed.[23] Other authors have also documented a learning curve for laparoscopic nephrectomy in terms of complication and conversion rates.[16,20]

## CONCLUSIONS

Laparoscopic nephrectomy has become a serious supplement to established operative techniques. The initial introduction has been followed by a period of consolidation, and finally standardization with good clinical results. Laparoscopic nephrectomy has to be considered as a clear winner over open nephrectomy with progressive technological development in laparoscopic surgery combined with routine practice over the years. Owing to its safety and reproducibility, laparoscopic nephrectomy is now the standard of care and should be offered to every patient with benign renal disease who is scheduled for elective nephrectomy.
